# Supplementary material for: Artificial intelligence in sepsis early prediction and diagnosis using unstructured data in healthcare
Source: Nat Commun. 2021 Jan 29;12:711. doi: 10.1038/s41467-021-20910-4 (PMC7846756; doi:10.1038/s41467-021-20910-4)
Supplement: Supplementary file 1 — Supplementary Information [file 41467_2021_20910_MOESM1_ESM.pdf]

Supplementary Information for "Artificial Intelligence in Sepsis Early Prediction and Diagnosis Using Unstructured Data in Healthcare"

Kim Huat Goh[1,4*], Le Wang[1,4*], Adrian Yong Kwang Yeow[2], Hermione Poh[3], Ke Li[3], Joannas Jie Lin Yeow[3], and Gamaliel Yu Heng Tan[3]

[1]Nanyang Business School, Nanyang Technological University, Singapore

[2]School of Business, Singapore University of Social Sciences, Singapore

[3]Academic Informatics Office, Medical Informatics, National University Health System, Singapore

[4]These authors contributed equally: Kim Huat Goh and Le Wang

[*]Correspondence to: akhgoh@ntu.edu.sg; lwang033@e.ntu.edu.sg

Overview

This document contains supplementary information relating to the paper. Supplementary Figures 1, 3 and 4 provide additional diagnostics of the SERA algorithm reported in the paper. Supplementary Figure 2 presents the detailed sampling frame used in this study.

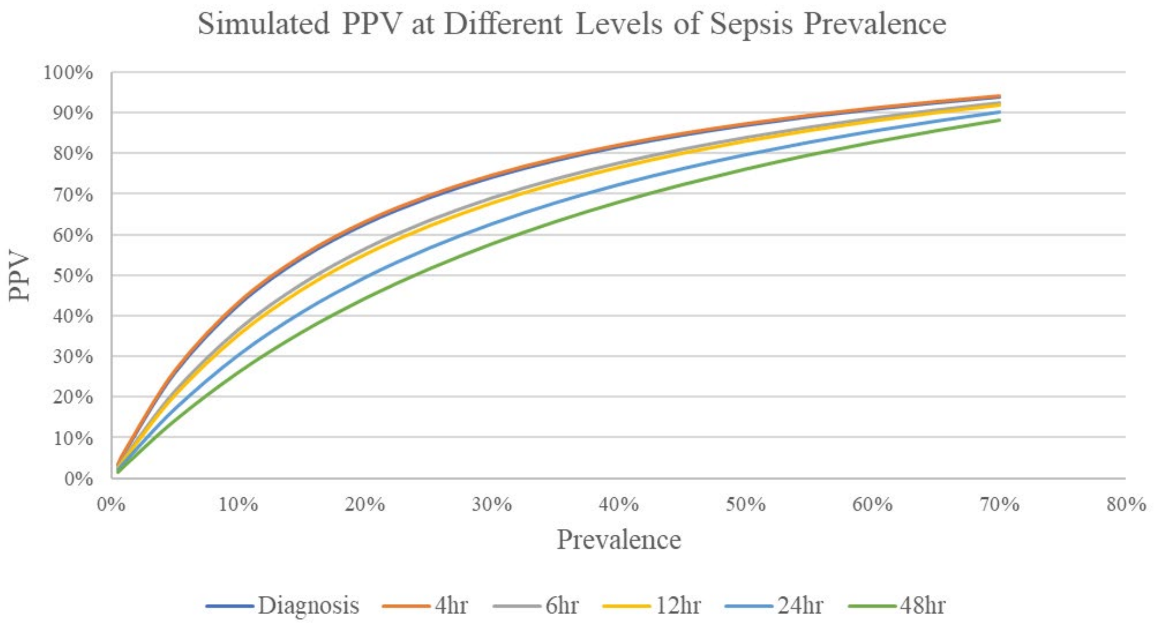

Supplementary Figure 1: Simulated PPV of SERA algorithm at Different Sepsis Prevalence Levels The chart shows how the PPV of SERA algorithm varies as sepsis prevalence changes.

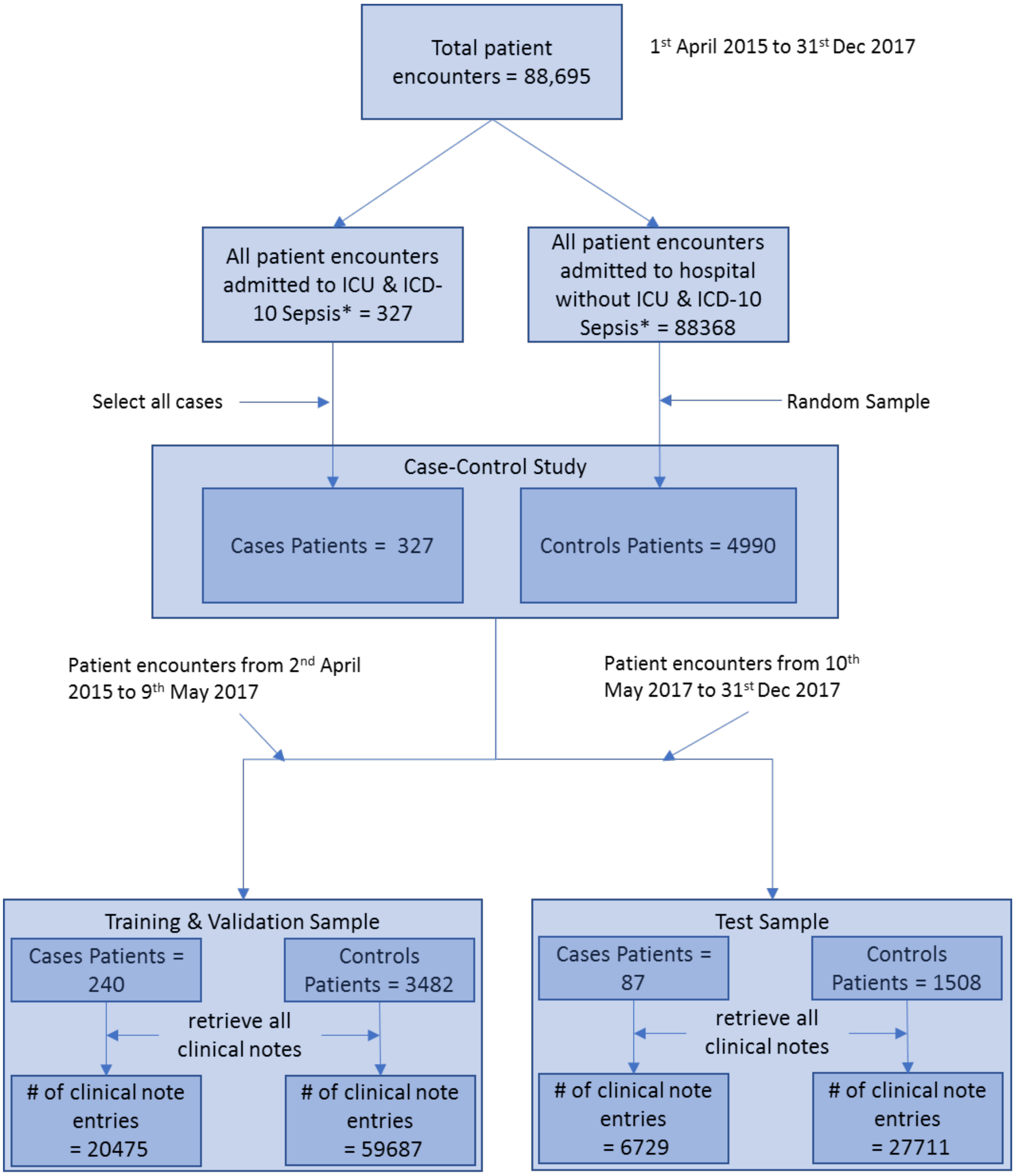

*** ICD-10 Code:**
Sepsis: 'A40.0','A40.1','A40.8','A40.9','A41.2','A41.0','A41.0Z16','A41.1','A40.3', 'A41.4','A41.50','A41.3','A41.51','A41.52','A41.53','A41.59','A41.81','A41.89', 'A41.9'
Severe Sepsis: 'R65.20','R65.21','R65.10','R65.11'

Supplementary Figure 2: Data Sample for Training, Validation, and Testing of SERA Algorithm

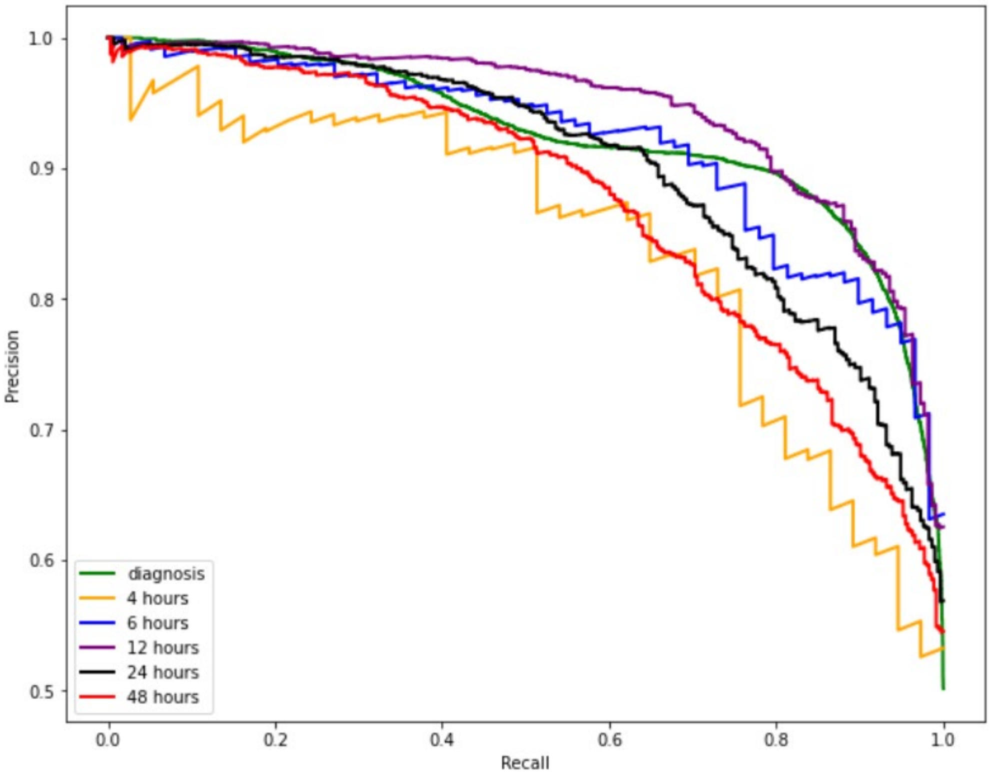

Supplementary Figure 3: Precision – Recall Curve (SERA algorithm). The vertical axis (precision) is truncated and starts at 0.5.

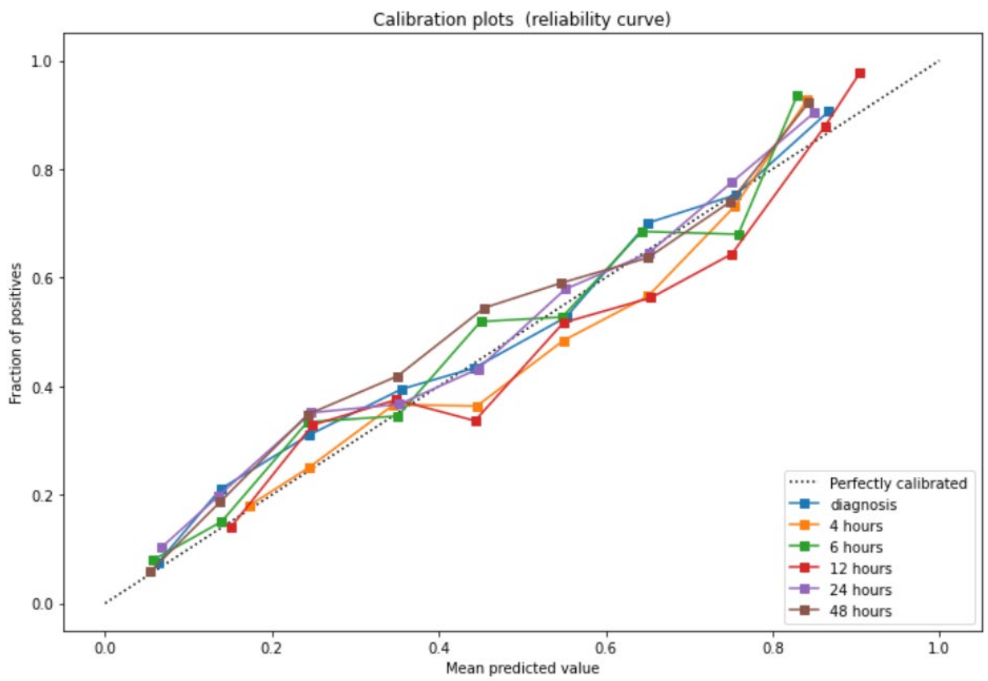

Supplementary Figure 4: Calibration Curves for SERA Algorithm
