# Supplementary material for: Artificial intelligence in sepsis early prediction and diagnosis using unstructured data in healthcare
Source: Nat Commun. 2021 Jan 29;12:711. doi: 10.1038/s41467-021-20910-4 (PMC7846756; doi:10.1038/s41467-021-20910-4)
Supplement: Supplementary file 3 — Reporting Summary [file 41467_2021_20910_MOESM3_ESM.pdf]

Corresponding author(s): Kim Huat Goh & Le Wang

Last updated by author(s): Dec 7, 2020

Reporting Summary

Nature Research wishes to improve the reproducibility of the work that we publish. This form provides structure for consistency and transparency in reporting. For further information on Nature Research policies, see Authors & Referees and the Editorial Policy Checklist .

Statistics

For all statistical analyses, confirm that the following items are present in the figure legend, table legend, main text, or Methods section.

n/a	Confirmed	
☐	☒	The exact sample size (*n*) for each experimental group/condition, given as a discrete number and unit of measurement
☐	☒	A statement on whether measurements were taken from distinct samples or whether the same sample was measured repeatedly
☒	☐	The statistical test(s) used AND whether they are one- or two-sided<br>*Only common tests should be described solely by name; describe more complex techniques in the Methods section.*
☐	☒	A description of all covariates tested
☐	☒	A description of any assumptions or corrections, such as tests of normality and adjustment for multiple comparisons
☐	☒	A full description of the statistical parameters including central tendency (e.g. means) or other basic estimates (e.g. regression coefficient) AND variation (e.g. standard deviation) or associated estimates of uncertainty (e.g. confidence intervals)
☒	☐	For null hypothesis testing, the test statistic (e.g. *F*, *t*, *r*) with confidence intervals, effect sizes, degrees of freedom and *P* value noted<br>*Give P values as exact values whenever suitable.*
☒	☐	For Bayesian analysis, information on the choice of priors and Markov chain Monte Carlo settings
☒	☐	For hierarchical and complex designs, identification of the appropriate level for tests and full reporting of outcomes
☒	☐	Estimates of effect sizes (e.g. Cohen's *d*, Pearson's *r*), indicating how they were calculated

Our web collection on statistics for biologists contains articles on many of the points above.

Software and code

Policy information about availability of computer code

Data collection	MySQL 8.0 was used to extract data from the EPIC(TM) Electronic Medical Record system.
Data analysis	SAS Enterprise Miner 14.2 was used to analyze the textual clinical notes and transform the free text into numerical matrices. The numerical matrices (from the clinical text) together with other patient vitals were analyzed using KNIME 4.1.6.

For manuscripts utilizing custom algorithms or software that are central to the research but not yet described in published literature, software must be made available to editors/reviewers. We strongly encourage code deposition in a community repository (e.g. GitHub). See the Nature Research guidelines for submitting code & software for further information.

Data

Policy information about availability of data

All manuscripts must include a data availability statement. This statement should provide the following information, where applicable:

- Accession codes, unique identifiers, or web links for publicly available datasets
- A list of figures that have associated raw data
- A description of any restrictions on data availability

The raw datasets generated and analyzed during the current study will not be published publicly due to privacy regulations under the Human Biomedical Research Act (HBRA) 2015 (Singapore). Raw datasets are available for review purposes. The raw data consists of clinical data from patients, including textual clinical notes written by physicians and contain information that could compromise research participant (patient) privacy or consent. The processed textual data with vitals is however available from the corresponding author on reasonable request.

Field-specific reporting

Please select the one below that is the best fit for your research. If you are not sure, read the appropriate sections before making your selection.

- [x] Life sciences
- [ ] Behavioural & social sciences
- [ ] Ecological, evolutionary & environmental sciences

For a reference copy of the document with all sections, see nature.com/documents/nr-reporting-summary-flat.pdf

Life sciences study design

All studies must disclose on these points even when the disclosure is negative.

Sample size	80,162 clinical notes from 3722 patients (training and validation sample). 34,440 clinical notes from 1,595 patients as independent test sample. Sample size was determined to be adequate based on the magnitude and consistency of measurable differences between groups. Sample contains all sepsis patients in the hospital between 2 April 2015 to 31 Dec 2017 together with randomly selected non-sepsis patients
Data exclusions	No data were excluded from the analyses.
Replication	Results was tested (replicated) using an independent sample of patients from a later date. The model was built and validated using patients admitted from 2 April 2015 to 9 May 2017. An identical specification of the model was tested again using a hold-out test sample of patients admitted to the hospital from 10 May 2017 to 31 Dec 2017. The replicated results are reported in the manuscript as the test results.
Randomization	Training and validation samples were randomly allocated, while the test sample was randomly selected from an independent, later study period.
Blinding	Blinding was not relevant to this study because group allocation was based on patient ID instead of concrete patient information.

Reporting for specific materials, systems and methods

We require information from authors about some types of materials, experimental systems and methods used in many studies. Here, indicate whether each material, system or method listed is relevant to your study. If you are not sure if a list item applies to your research, read the appropriate section before selecting a response.

Materials & experimental systems

n/a	Involved in the study	
x		Antibodies
x		Eukaryotic cell lines
x		Palaeontology
x		Animals and other organisms
	x	Human research participants
x		Clinical data

Methods

n/a	Involved in the study	
x		ChIP-seq
x		Flow cytometry
x		MRI-based neuroimaging

Human research participants

Policy information about studies involving human research participants

Population characteristics	Patients admitted into Ng Teng Fong General Hospital. Sample consists of 5,317 patients admitted from 2 April 2015 to 31 Dec 2017.<br><br>In Training and Validation Sample<br>Number of patients 3722<br>Age - years old 63.71 ± 17.08 (Mean ± SD)<br>Male - % 57.3<br>Length of hospital stay - days 5.52 ± 14.31 (Mean ± SD)<br>ICU Admission - % 7.52<br>Mortality - % 4.5<br>Septic - % 6.45%<br>Non-septic - % 93.55%<br><br>In Test Sample (replication)<br>Number of patients 1595<br>Age - years old 63.90±16.81 (Mean±SD)<br>Male - % 60.67<br>Length of hospital stay - days 5.17±10.80 (Mean±SD)<br>ICU Admission - % 8.61<br>Mortality - % 5.01<br>Septic - % 5.45%<br>Non-septic - % 94.55%
Recruitment	Treatment Group: All patients encounters admitted to ICU & with ICD-10 Code Sepsis: 'A40.0','A40.1','A40.8','A40.9','A41.2','A41.0','A41.0Z16','A41.1','A40.3', 'A41.4','A41.50','A41.3','A41.51','A41.52','A41.53','A41.59','A41.81','A41.89', 'A41.9' Severe Sepsis: 'R65.20','R65.21','R65.10','R65.11' from 2 April 2015 to 31 Dec 2017<br>Control Group: Random sample of 4990 patient encounters from 88,368 hospital admission between 2 April 2015 to 31 Dec 2017. A retrospective chart review was conducted with this data.
Ethics oversight	National Healthcare Group (NHG) Domain Specific Review Board (DSRB). Reference 2018/00455. Approved 26 July 2018

Note that full information on the approval of the study protocol must also be provided in the manuscript.
